# Detection of Adenoviral *E1A* Gene in Guthrie Cards for Insights into Pediatric Cancer Origin

**DOI:** 10.3390/ijms27094047

**Published:** 2026-04-30

**Authors:** Gracia Mendoza, Rebeca Guerrero, Mark Strunk, Carlota Calvo, Yolanda González-Irazabal, Ramiro Álvarez, Jorge E. Gomez-Sirvent, Ricardo López-Almaraz, Javier Hernández-Losa, Santiago Ramón y Cajal, Rebeca González-Pastor, Pilar Martin-Duque

**Affiliations:** 1 Instituto de Investigación Sanitaria Aragón (IIS Aragón), 50009 Zaragoza, Spain; gmendoza@iisaragon.es; 2 Department of Pharmacology and Physiology, Forensic and Legal Medicine, Veterinary Faculty, University of Zaragoza, 50013 Zaragoza, Spain; 3 Instituto Aragonés de Ciencias de la Salud (IACS Aragón), 50009 Zaragoza, Spain; rebeg@unizar.es (R.G.); mhpstrunk.iacs@aragon.es (M.S.); 4 Departments of Pediatric Oncology, Clinical Biochemistry and Pathology, Miguel Servet University Hospital, 50009 Zaragoza, Spain; ccalvoes@salud.aragon.es (C.C.); yolanda.gonzalezi@sespa.es (Y.G.-I.); ralvarezal@gmail.com (R.Á.); 5 Department of Pediatrics, Hospital Universitario Nuestra Señora de Candelaria, 38010 Santa Cruz de Tenerife, Spain; jgomsir@gobiernodecanarias.org; 6 Pediatric Oncology-Hematology Unit, Hospital Universitario Cruces, BioBizkaia Health Research Institute, 48903 Barakaldo, Spain; ricardo.lopezalmaraz@osakidetza.eus; 7 Department of Pathology, Hospital Universitari Vall d’Hebron (HUVH), Campus Vall d’Hebron, Passeig Vall d’Hebron 119-129, 08035 Barcelona, Spain; javier.hernandez@vallhebron.cat (J.H.-L.); santiago.ramonycajal@vallhebron.cat (S.R.y.C.); 8 Translational Molecular Pathology, Vall d’Hebron Research Institute (VHIR), Universitat Autonoma de Barcelona (UAB), Passeig Vall d’Hebron 119-129, 08035 Barcelona, Spain; 9 Spanish Biomedical Research Network Centre in Oncology (CIBERONC), Av. Monforte de Lemos 3-5, 28029 Madrid, Spain; 10 Centro de Investigación Biomédica (CENBIO), Facultad de Ciencias de la Salud Eugenio Espejo, Universidad UTE, Quito 170527, Ecuador; 11 Nanomedicines and Nanotherapies Unit, Department of Development of Medicines and Advanced Therapies, Instituto de Salud Carlos III, 28222 Majadahonda, Spain

**Keywords:** adenovirus, cytomegalovirus, Guthrie cards, dried blood spots, non-invasive diagnostics, viral oncogenesis, pediatric oncology, early cancer detection, Sustainable Development Goal 3

## Abstract

Adenoviruses have been implicated in childhood cancers, primarily leukemia, yet prior neonatal investigations have rarely examined other pediatric tumor types. This study evaluated whether adenoviral early region (*E1A*) sequences can be detected in archival neonatal Guthrie cards from children who later developed diverse pediatric tumors and in corresponding paraffin-embedded tissues. DNA extraction was optimized for long-stored dried blood spots, and PCR conditions were refined for both Guthrie card and paraffin-derived DNA. Adenoviral *E1A* was analyzed using conventional and nested PCR, and sequencing of representative amplicons confirmed correspondence to human adenovirus species C. *E1A* PCR positivity was found in 43% of Guthrie cards from cases (n = 54) and 34% of controls (n = 32), and in 41% of tumor tissues (n = 75) compared with 5% of non-tumor paraffin controls (n = 20). Detection occurred across multiple tumor categories without a clear association with tumor type. Partial concordance was observed between paired neonatal and tumor samples, and cytomegalovirus markers were detected in a subset of *E1A*-positive specimens. These findings confirm the suitability of Guthrie cards for retrospective viral DNA detection and extend previous leukemia-focused neonatal studies to broader pediatric tumors. The data suggest a potential association between birth-stage adenoviral detection and childhood cancer, though a causal link remains unproven and requires further longitudinal investigation.

## 1. Introduction

Infectious agents have long been recognized as important contributors to cancer pathogenesis [1]. Several viruses are linked to oncogenesis either through immune suppression or by directly inducing oncogene-mediated cellular transformation [2,3]. Examples include human T-lymphotropic virus type 1 in acute leukemia, human papillomavirus (HPV) in cervical carcinoma, hepatitis B virus in hepatocellular carcinoma, and Epstein–Barr virus in Burkitt’s lymphoma [4,5,6,7]. Epidemiological evidence supports a possible viral involvement in pediatric tumors, although molecular confirmation in most pediatric cancers remains lacking [8,9]. 

Among these viral contributors, adenoviruses have attracted particular attention. Although adenoviral infections are typically mild and confined to the upper respiratory tract, they occur primarily in early childhood and can establish persistence in tonsillar or peripheral blood lymphocytes [10,11,12]. This early and persistent presence in lymphoid tissues raises the possibility of a contributory role in tumor initiation, aligning with current etiological models of childhood leukemia that emphasize prenatal and early-life exposures. 

Experimental and observational studies have suggested oncogenic potential for some adenoviruses, including reports involving adenoviral DNA detection in human lung carcinoma and tumor induction by adenovirus type 9 and type 12 in animal models [13,14,15,16]. However, the biological consequences of adenoviral infection depend on tissue and host context. Human adenoviruses commonly infect mucosal epithelial tissues, especially the upper respiratory tract, but also the lower respiratory tract, conjunctiva, and gastrointestinal tract, with type-specific differences in tropism [12]. In these settings, adenoviruses typically establish productive lytic infection, whereas experimental transformation has been demonstrated mainly in non-permissive systems in which viral replication is restricted or abortive [17,18]. The adenoviral early region 1A (E1A) protein plays a pivotal mechanistic role by interacting with tumor-suppressor proteins such as retinoblastoma (Rb), thereby disrupting normal cell-cycle regulation and transcriptional control [19]. Within adenoviral transformation models, these effects occur in a broader early-gene context that includes E1B and some E4 gene products, which contribute to the transforming phenotype by modulating p53-dependent responses, apoptosis, and cell survival [19,20]. Together, these observations support biological plausibility for a possible contribution of adenoviral early gene activity to oncogenic processes. Nevertheless, molecular detection of adenoviral sequences in human tumors has been inconsistent [21]. 

Prenatal viral exposures, including species C adenoviruses, Epstein–Barr virus, and polyomaviruses, have been investigated as potential early “first hits” in leukemogenesis [22,23,24]. Adenoviral DNA has been detected in a minority of neonatal samples, but current evidence does not establish a causal relationship with childhood leukemia or other cancers [8,21]. The proposed “hit-and-run” model of viral oncogenesis, whereby transient infection initiates oncogenic changes without persistent viral presence, therefore remains hypothetical [25]. Supporting this concept, cytokine profiling at birth has revealed altered immune signatures in children who later developed acute lymphoblastic leukemia, suggesting that prenatal immune dysregulation may interact with early viral exposures to influence disease susceptibility [26]. 

Beyond leukemias [8,21,27,28], few studies have explored whether adenoviral sequences might also be associated with other pediatric malignancies, such as central nervous system, hepatic, germ-cell, or soft-tissue tumors [29]. Moreover, the majority of available reports rely on either neonatal blood spots or tumor tissues alone, rarely analyzing both within the same individual. This limits our understanding of whether adenoviral DNA detected at birth persists, disappears, or reappears during tumorigenesis. Clarifying whether molecular traces of adenoviral infection can be identified at birth and later correlated with tumor development is, therefore, essential, as prenatal or maternally transmitted viral exposures may contribute to genomic instability and early oncogenic risk [24,30]. 

To address these questions, we investigated the presence of adenoviral *E1A* gene sequences in neonatal blood samples collected on Guthrie cards and in DNA extracted from paraffin-embedded pediatric tumors. Whenever possible, we compared neonatal and tumor DNA from the same individuals and included matched benign controls. The study was based on the premise that adenoviral *E1A* sequences might be detectable at birth in children who later developed pediatric tumors and might also be present in corresponding tumor tissues. Determining whether adenoviral DNA was already detectable at birth in children who later developed cancer represents an important step toward understanding the temporal relationships between early-life viral exposure and pediatric oncogenesis. Such efforts may contribute to the longer-term goal of identifying early-life markers relevant to earlier diagnosis and improved outcomes, particularly in resource-limited settings. 

## 2. Results

Archival neonatal Guthrie cards [31,32] and paraffin-embedded pediatric tumor specimens were analyzed for the presence of adenoviral *E1A* sequences following optimization of DNA extraction and assessment of DNA integrity and amplification performance. 

To enable reliable molecular analysis, multiple DNA extraction strategies were systematically explored to optimize DNA recovery from Guthrie cards (Figure S1), which contain very limited amounts of dried blood and are known to present challenges due to extended storage and nucleic acid degradation. As neonatal screening cards are often stored for years at ambient temperature, oxidative and hydrolytic damage could substantially reduce the yield of amplifiable DNA [33,34,35]. Methods based on the Whatman FTA BD09 system (GE Healthcare, Little Chalfont, UK) and Chelex 100 resin (Bio-Rad, Hercules, CA, USA) were tested, but consistently produced suboptimal DNA yields for downstream analysis [36]. To compensate for the low recoveries, a post-extraction whole-genome amplification step using the GenomiPhi kit (Amersham Biosciences, Little Chalfont, UK) was evaluated; however, this multiple-displacement amplification (MDA)-based approach introduced amplification bias and failed to increase the amount of quantifiable template DNA [37,38]. A modified QIAamp DNA Mini Kit protocol was ultimately adopted as the most reliable and reproducible method [39]. 

Most Guthrie cards analyzed had been stored at 4 °C for up to two decades, and several showed marked physical deterioration, posing challenges for DNA recovery. Nevertheless, high-quality DNA was successfully recovered from nearly all samples, with yields and purity values consistent with those summarized in Table 1. These results align with previous reports indicating A260/A280 ratios of 1.7–2.0 and DNA concentrations ranging from approximately 2–24 ng/µL, consistent with quantities reported to be sufficient for PCR-based downstream applications [40,41]. In comparison, DNA obtained from paraffin-embedded tissues exhibited markedly greater variability in both concentration and A260/A280 ratios (Table 1), reflecting the combined effects of fixation-induced crosslinking, protein contamination, and storage duration, factors known to compromise DNA integrity and optical purity [41,42]. Overall, both the Guthrie card and paraffin-derived DNA were suitable for subsequent PCR analyses, despite differences in yield and integrity. 

**Figure 1:**
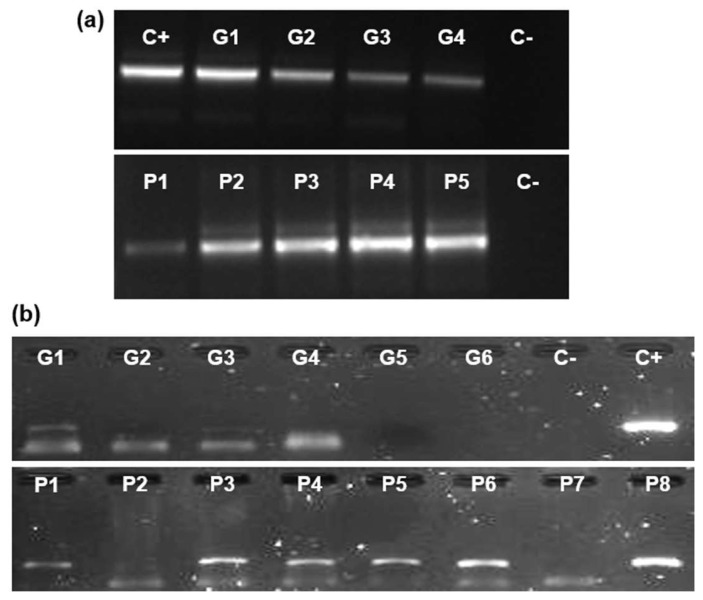
PCR amplification in Guthrie cards and paraffin-derived DNA. (**a**) *β-actin* amplification confirming the presence of amplifiable DNA in all samples (amplicon size: 828 bp). (**b**) Detection of *E1A* by nested PCR (amplicon size: 208 bp). Representative samples are shown for both panels. Lanes labeled G correspond to Guthrie card samples (G1, G2, etc.), and lanes labeled P to paraffin-derived samples (P1, P2, etc.). *E1A*-positive samples: G1, G3, P1, P3, P4, P5, P6, P8. *E1A*-negative samples: G2, G4, G5, G6, P2, and P7. C^+^: positive control (293 cell DNA for *β-actin*; template-spiked reaction for *E1A*). C^−^: water negative control.

To confirm that the recovered DNA was suitable for downstream PCR analysis, amplification of the *β-actin* housekeeping gene was performed in all samples. *β-actin* amplification was successful in all cases, confirming that despite minor yield variation and partial degradation, the extracted DNA remained amplifiable and suitable for subsequent *E1A*-targeted PCR assays (Figure 1a).

Subsequent PCR assays were performed to detect adenoviral *E1A* sequences. Primer sets and cycling conditions were optimized to account for variability in DNA quality from Guthrie cards and paraffin-embedded tissues. Two primer sets were evaluated for conventional PCR: one amplifying a longer fragment (383 bp), which was effective with Guthrie card DNA but not with DNA from paraffin-embedded tissues, and another targeting a shorter fragment (148 bp), which amplified both sources, though inconsistently in paraffin-derived samples (Figure S2). To improve detection, nested PCR targeting a 208 bp region of *E1A* was employed, yielding greater sensitivity and reproducibility than conventional PCR across both sample types (Figure 1b). Positive controls, including 293 cells and template-spiked reactions diluted to match sample concentrations, consistently amplified, while extraction blanks and no-template controls remained negative in all runs, confirming the performance of the assay controls during extraction and amplification. However, potential contamination between Guthrie cards during sample storage cannot be fully excluded. 

Sanger sequencing was performed on 23 PCR-positive amplicons with adequate product quality. Among these, adenoviral sequences were confirmed in 9 of 11 Guthrie card samples and 5 of 12 paraffin-derived samples. The confirmed sequences were identical or nearly identical across positive samples; therefore, a representative non-redundant consensus sequence corresponding to the internal region of the *E1A* amplicon was used for database comparison. BLAST analyses identified top matches to *Human mastadenovirus C* members, including types 1, 2, 5, 6, 57, 89, and 108, with 100% identity over the aligned region. No significant homology to the human genome was observed (Table S1). 

Based on PCR detection, *E1A* frequencies were evaluated in patient-derived samples and matched controls (Table 2). Patient-derived Guthrie cards showed 43% *E1A* positivity, whereas 34% of control Guthrie cards were positive (not statistically significant, *p* = 0.5002). In paraffin-embedded tissues, *E1A* was detected in 41% of tumor samples compared with 5% of paraffin controls (statistically significant, *p* = 0.0026).

**Figure 2:**
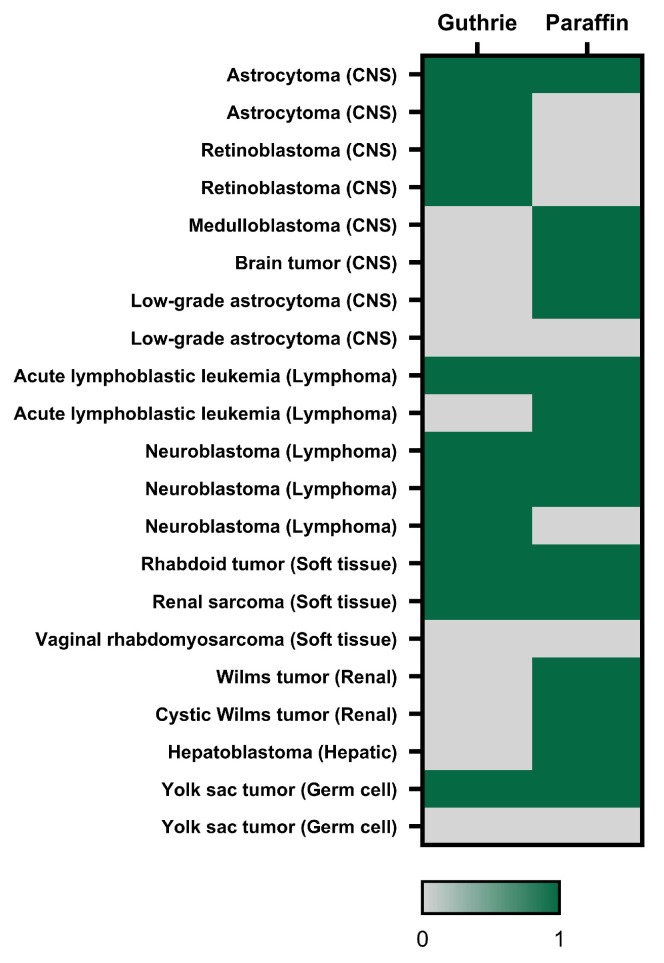
Patient-level distribution of *E1A* detection in paired Guthrie cards and tumor samples. Each row represents one patient with a matched neonatal Guthrie card and paraffin-embedded tumor sample (n = 21). *E1A* positivity or negativity is shown for both sample types. Tumors are grouped as central nervous system, lymphoma, soft tissue, renal, hepatic, and germ cell tumors.

To further examine whether neonatal *E1A* status was concordant between neonatal and tumor samples, paired analysis of Guthrie cards and paraffin tumors was performed in cases where both sample types were available. Among 32 initially eligible patients, 21 complete paired samples were available due to the unavailability of paraffin blocks for 11 cases. Among these paired cases, 7 individuals were positive in both sample types (7/21, 33%) and 3 were negative in both (3/21, 14%), demonstrating concordant *E1A* status in 10 cases (10/21, 48%). In contrast, 4 patients showed *E1A*-positive Guthrie cards with negative paraffin tumors (4/21, 19%), whereas 7 exhibited the opposite pattern, with *E1A*-negative Guthrie cards but positive paraffin tumors (7/21, 33%). Overall, paired analysis showed limited agreement between neonatal and tumor samples. To better visualize concordance and discordance patterns across paired samples, these relationships are summarized in Figure 2. Although *E1A* detection was somewhat more frequent in tumor-only discordant pairs than in Guthrie-only discordant pairs, this difference was not statistically significant (*p* = 0.54). 

**Figure 3:**
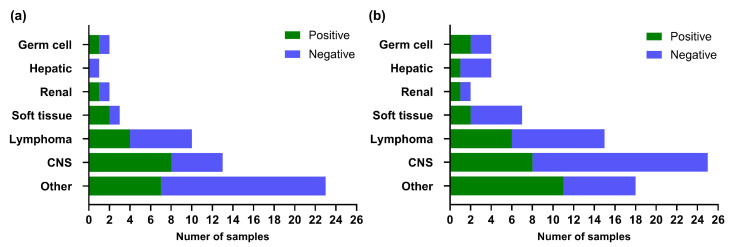
Number of *E1A* positive and negative samples by tumor type. Bars show the distribution of *E1A* detection in (**a**) Guthrie cards and (**b**) paraffin tumor samples. Tumor types were grouped by tissue of origin; detailed classifications are provided in the Table S2.

We next examined the distribution of *E1A* detection across tumor types in both Guthrie cards and paraffin-embedded tumors (Figure 3). Among the *E1A*-positive Guthrie card samples, central nervous system (CNS) tumors were numerically most represented, while lymphomas showed a more variable pattern. Soft tissue and hepatic tumors yielded fewer cases overall but still included positives, whereas germ cell tumors showed a balanced distribution. The “Other” category, comprising diverse tumor sites, also included positive cases. In paraffin samples, the overall distribution was broader but generally consistent across categories. CNS tumors presented both positive and negative cases, and lymphomas again showed mixed results. Soft tissue tumors were predominantly negative, whereas hepatic and germ cell tumors each showed occasional positives. Notably, the “Other” group displayed a relatively high proportion of positives, reflecting its heterogeneous tumor composition. 

Taken together, *E1A* sequences were detected across multiple tumor types without a clear predilection for any specific category. Moreover, no statistically significant association was observed between *E1A* detection and diagnostic category in Guthrie card samples (*p* = 0.2757) or in paraffin tumor samples (*p* = 0.4349), suggesting that *E1A* detection was not confined to a specific tumor category in this cohort. 

To explore whether *E1A* detection showed temporal clustering, birth dates and tumor types were analyzed across the cohort. No clear temporal clustering was observed, and patients displayed a broad distribution of tumor categories, with leukemias and central nervous system tumors recurring throughout the study period. Leukemias appeared slightly more frequent in certain birth groups, likely reflecting random sampling rather than a defined cohort effect. Overall, no association between year of birth and tumor type was observed. When focusing specifically on *E1A*-positive Guthrie cards, several positive cases were observed within a relatively narrow birth-date interval. However, *E1A*-positive cases were also present outside this interval, indicating that positivity was not restricted to a single temporal cluster. 

Overall, *E1A* sequences were detected in both neonatal Guthrie cards and paraffin-embedded tumor samples across multiple pediatric tumor types, with limited concordance between paired neonatal and tumor specimens. 

## 3. Discussion

Adenoviruses remain relevant candidates in pediatric oncogenesis because of their early-life exposure, capacity for persistence [43,44,45,46], and the transforming properties of the *E1A* gene, which can dysregulate cell-cycle progression and host gene expression, are processes central to malignant transformation [47]. However, because detection of viral DNA alone does not establish causality, its interpretation requires careful consideration of the timing and tissue context in which viral sequences are identified. In this context, evaluating whether adenoviral sequences are detectable at birth and later in tumor tissues represents an exploratory approach to assessing temporal relationships between early-life viral exposure and later pediatric tumor development. The present findings demonstrate that adenoviral *E1A* sequences can be recovered from long-stored neonatal Guthrie cards and detected in a subset of paraffin-embedded pediatric tumors, supporting the utility of these archival materials for retrospective molecular assessment of early-life viral exposures and tumor-associated viral DNA [48,49]. *E1A* detection in paraffin-embedded tumors, obtained after diagnosis, reflects the presence of viral DNA within established disease tissues but does not necessarily indicate persistence of a neonatal infection [1]. Thus, combined neonatal and tumor analysis allows temporal comparison of *E1A* detection without implying continuous viral persistence. 

The successful recovery of amplifiable DNA from long-stored Guthrie cards was supported by DNA concentration and purity values within ranges reported to be compatible with PCR-based downstream applications. Although variation in yield and purity was observed, likely reflecting differences in blood spot quality, storage duration, and nucleic acid degradation, it did not prevent the recovery of amplifiable DNA. These findings underscore the long-term stability of dried blood spots and support their suitability as archival material for retrospective molecular analyses, even after extended storage under non-ideal archival conditions. In contrast, DNA recovered from paraffin-embedded tissues showed greater variability, as expected for formalin-fixed paraffin-embedded (FFPE)-derived material, but remained sufficiently amplifiable for targeted PCR analysis. Successful *β-actin* amplification and assay-control performance reduce the likelihood that *E1A* detection patterns were driven solely by global PCR failure. Nevertheless, differences in DNA preservation, tissue composition, and template availability remain important factors when interpreting *E1A* detection across neonatal cards, tumor tissues, and controls. 

Detection of *E1A* sequences in neonatal Guthrie cards in both patient-derived and control samples indicates that adenoviral DNA can be identified at birth, but the modest and non-significant difference between groups argues against a clear case-enriched association in this cohort. *E1A* detection in neonatal samples, including controls, may reflect background adenoviral circulation, transient perinatal exposure, maternal or environmental viral contact, or low-level viral persistence without evident pathological consequence. Because no longitudinal follow-up exists for control individuals, it cannot be determined whether some may later develop malignancy. These findings therefore support the technical feasibility of neonatal *E1A* detection while emphasizing the need for cautious interpretation of case–control differences. The absence of amplification in negative controls supports run-level assay specificity, although pre-analytical contamination or cross-contact during long-term card storage cannot be fully excluded. 

In contrast to the neonatal samples, *E1A* detection in paraffin-embedded tumor tissues showed a clearer difference between malignant and control specimens. The rarity of positive findings among controls supports the specificity of detection in tumor samples, though sporadic amplification in non-tumor tissues suggests that low-level adenoviral DNA can occasionally be detected outside malignant contexts. However, the low *E1A* prevalence observed in paraffin controls may partly reflect the limited and predominantly tonsillar composition of the normal tissue group, since tissue-specific features, as well as differences in tissue composition and preservation between tumor and non-tumor samples, can influence viral DNA detectability, rather than only a true absence of viral DNA in non-tumor tissues. Therefore, the higher frequency of *E1A* detection in tumor tissues is an important observation, but it should not be interpreted as direct evidence of causality without validation in larger, tissue-matched cohorts.

The paired analysis further illustrates the complexity of interpreting *E1A* detection across time and tissue compartments. If neonatal adenoviral DNA detected at birth were consistently maintained during tumor development, stronger concordance between Guthrie cards and corresponding tumors would be expected. Instead, the limited agreement observed between paired samples suggests that *E1A* detectability may vary according to biological compartment, timing of sampling, viral copy number, and technical constraints of archival DNA analysis. Cases positive in both sample types may reflect continued detectability of adenoviral DNA, repeated exposure, or independent detection of related adenoviral sequences across time; whereas Guthrie-positive/tumor-negative cases may indicate transient neonatal exposure followed by viral clearance, viral levels below detection in tumor tissue, or absence from the sampled tumor region. Conversely, tumor-positive/Guthrie-negative cases may reflect later acquisition, tissue-specific enrichment, or neonatal viral levels below the detection threshold. Because the difference between discordant patterns was not statistically significant, these interpretations remain exploratory. Overall, the paired results argue against a uniform model of continuous viral persistence from birth to tumor diagnosis and instead support a more heterogeneous scenario involving exposure, clearance, persistence, and tissue-specific detectability.

Together, the neonatal, tumor, and paired-sample findings indicate that *E1A* detection is not uniform across biological compartments or time points. Guthrie cards reflect viral DNA detectable at birth, whereas paraffin-embedded tumors represent established disease tissue obtained after diagnosis. Therefore, differences between these materials should be interpreted in relation to sample origin, tissue composition, DNA preservation, and assay sensitivity, rather than as direct evidence of continuous viral persistence from birth to tumor development. 

Contextualizing our Guthrie-card findings with prior attempts is essential. Earlier neonatal dried blood spot (DBS) studies reported an association between adenoviral DNA and later childhood ALL (e.g., 26.5% in cases vs. 6.4% in controls) but these findings were later challenged by studies that did not observe a similar excess and by evidence that very low viral copy numbers, limited infected-cell frequency and assay-sensitivity constraints complicate reliable detection [8,27,28,50]. Compared with those reports, our control positivity (34%) was higher, and case positivity (43%) showed only a modest separation from controls. This discrepancy likely reflects differences in extraction from aged DBS, nested-PCR sensitivity, pre-analytical sample handling, contamination-control procedures, and population-level circulation and persistence of human mastadenovirus species C (HAdV-C) in pediatric lymphoid tissues [51,52,53,54]. Accordingly, our data indicate feasibility of neonatal *E1A* detection but argue for cautious interpretation of case–control contrasts when baseline community exposure is high and templates are limited. 

Unlike most prior newborn-spot studies that focused solely on childhood ALL, our analysis spans diverse pediatric tumor types, including CNS tumors, lymphomas, soft tissue tumors, hepatic tumors, and germ-cell tumors, thereby broadening the clinical context in which neonatal and tumor-associated *E1A* detection can be examined. Mechanistically, adenovirus can persist in tonsils/adenoids and reactivate, providing a plausible source of low-level DNA detectable in later specimens [12,54,55,56]. While our data do not establish timing, persistence, or causation, they reinforce the feasibility of using archived neonatal blood spots as a molecular window into early-life events that may be relevant to tumorigenesis. 

Experimental models have demonstrated that adenovirus infection can profoundly alter host–cell regulation. Specifically, the adenoviral E1A protein engages the histone acetyltransferases p300 and CREB-binding protein (p300/CBP) and drives broad transcriptional reprogramming via Rb/E2F-linked regulatory networks, impacting both cell-cycle and innate/immune-response gene programs [57,58,59,60,61]. These virus-driven chromatin alterations may affect long-term cellular programming and differentiation potential [62]. Concurrently, early adenoviral transcription is subject to host chromatin-based restriction: the immediate-early *E1A* promoter can be repressed by death-domain associated (Daxx) protein and the Daxx/alpha-thalassemia/mental retardation syndrome X-linked (ATRX) protein axis, providing a mechanistic basis for epigenetic silencing, very low viral copy number, and intermittent PCR detectability in clinical specimens [57,63,64]. Accordingly, transient early *E1A* exposure could perturb host regulatory programs while leaving intermittent or undetectable *E1A* signals in Guthrie cards or tumors at later time points [65]. If such events occurred during prenatal or perinatal infection, the resulting molecular imprints could persist in neonatal tissues and contribute to later malignant transformation. Although this study did not directly assess epigenetic endpoints and PCR detection of *E1A* alone does not provide evidence for these mechanisms, prior reports in pediatric tumors describe atypical DNA-methylation and histone-modification patterns that parallel virus-induced chromatin dysregulation [66,67,68]. 

The modest temporal grouping observed among *E1A*-positive Guthrie cards may support the interpretation of episodic population exposure rather than a persistent birth-cohort effect. Although tumor occurrence itself showed no clear temporal structure, several positive neonatal samples clustered within a relatively narrow birth period. This pattern may reflect shared gestational or perinatal exposure windows consistent with transient adenoviral circulation, although positive cases were also observed outside this interval [69,70]. Therefore, the temporal distribution does not define a discrete outbreak or cohort effect, but it is compatible with intermittent population-level viral circulation.

These temporal observations are consistent with the broader heterogeneity observed across neonatal cards, tumor tissues, and paired samples. Concepts such as hit-and-run oncogenesis and multistep models remain biologically plausible based on prior experimental literature [18,25,30,71]. These observations, when integrated with experimental data on E1A–chromatin interactions, raise the possibility that viral persistence or reactivation could have downstream epigenetic consequences [72], although this was not assessed in the present study. 

In addition, prior analyses on a subset of Guthrie cards had detected additional viral markers, including cytomegalovirus (CMV), simian virus 40 (SV40), human papillomavirus, and herpesvirus. Approximately half of the *E1A*-positive samples were also CMV-positive, without clear tumor-type association. These data are consistent with the notion that CMV exposure may act as an additive or sequential perinatal event [53,73], reinforcing the hypothesis that multiple early-life viral infections could cumulatively contribute to molecular alterations relevant to tumor susceptibility or later disease development [74,75,76,77]. Viewed in this context, neonatal *E1A* detection may represent part of a broader early-life viral exposure landscape rather than the action of a single etiologic virus. Co-detection does not necessarily indicate persistent co-infection; it may reflect overlapping windows of early-life exposure and immune activation that could act as a modifying or promoting context for later disease development. However, the present data do not establish whether adenoviral exposure acts independently, interacts with other viral infections, or simply reflects background early-life viral contact. This distinction is important because models such as multistep or “hit-and-run” viral oncogenesis require temporal, quantitative, and mechanistic evidence that cannot be inferred from PCR detection alone. Together, these findings highlight the biological complexity underlying viral detection patterns and set the context for evaluating the technical scope and limitations of this study. 

Given the variable and often low-level detectability of viral sequences in archival material, the technical strengths and study limitations are central to interpreting these findings. Strengths include optimized DNA recovery from aged DBS and FFPE samples, use of short-amplicon nested PCR, inclusion of diverse tumor types, and availability of benign pediatric controls and paired Guthrie–paraffin samples in several cases. Limitations include archival specimen age, possible DNA degradation, lack of quantitative viral load or transcription data, incomplete sequencing confirmation of all PCR-positive amplicons, and incomplete pairing of neonatal and tumor samples. Moreover, the presence of viral DNA alone does not establish causation, and prior DBS studies, including those targeting *E1A*, have yielded inconsistent case–control differences [8,27,28], emphasizing the need for cautious interpretation. The qualitative nature of nested PCR also limits conclusions regarding viral burden, clonality, cellular localization, and biological activity. 

In summary, our results show that *E1A* sequences are recoverable from neonatal Guthrie cards and can also be detected in a subset of paraffin-embedded tumors, that *E1A* detection is moderately higher in patient-derived material than in controls, and that concordance between neonatal and tumor specimens is limited. These findings support a possible association between early-life *E1A* presence and childhood cancer development, while acknowledging uncertainty about timing, variability, and the biological meaning of detection. Further quantitative and longitudinal studies integrating viral, genetic, and epigenetic data will be essential to clarify whether *E1A* detection marks transient exposure, low-level or episodic persistence, or an early biological signal associated with childhood cancer risk. These findings position Guthrie cards as a valuable resource for probing early viral-exposure events and highlight that the link between adenovirus and childhood cancer is still unresolved. 

## 4. Materials and Methods

### 4.1. Patients

Children up to 4 years who later developed cancer were randomly identified by Servicio de Oncología Pediátrica y Laboratorio de Genética del Hospital Universitario Miguel Servet (Zaragoza, Spain), and by Servicio de Genética Pediátrica del Hospital de la Candelaria and Hospital de la Laguna (Tenerife, Spain). Additional paraffin-embedded tumor samples were obtained from pathology archives at Vall d’Hebron Hospital (Barcelona) and Miguel Servet University Hospital (Zaragoza) for complementary analyses. Controls, matching for age and birthplace, were also collected for further comparisons. Guthrie cards and paraffin tissues corresponded to partially overlapping but not identical subsets of the study population. 

In total, 54 Guthrie card samples from children who later developed cancer and 32 Guthrie card samples from cancer-free controls were analyzed. In addition, 75 paraffin-embedded tumor tissues and 20 paraffin control tissues were included. Sample numbers were constrained by the presence and condition of Guthrie cards in neonatal archives and of paraffin blocks in pathology repositories. The median age at cancer diagnosis for these patients was 5 years (2 months–15 years) between 1997 and 2009. Diagnoses included hematological malignancies, solid tumors, and other pediatric cancers, reflecting a heterogeneous cohort. 

All samples were fully anonymized prior to receipt in our laboratory; therefore, no identifiable personal data were processed. The study protocol, including procedures for obtaining Guthrie cards and extracting DNA from paraffin-embedded tissues, was submitted to the Research Ethics Committee of Aragón (CEICA) and conducted under reference PI09/090. 

### 4.2. Sample Collection

DBS collected 1–4 days after birth for genetic screening were analyzed from children who later developed cancer, selected from those diagnosed up to 4 years of age. Each sample consisted of capillary blood taken from the heel of neonates, blotted onto filter paper (Guthrie cards), dried, and stored at 4 °C. The median storage time for the DBS samples was 8.5 years (range: 2–14 years). Uniform 5 mm discs were cut from each Guthrie card using a sterile puncher, with new blades for each sample to avoid cross-contamination. 

Paraffin-embedded tissue samples comprised both tumor and benign pediatric specimens (mostly tonsils and other benign pediatric specimens) from children up to 15 years old. To minimize contamination, the first few sections of each block were discarded, and microtome blades were cleaned between samples. 

### 4.3. DNA Extraction

Uniform 5 mm discs were excised from each Guthrie card using sterile punchers, with new blades for each sample to prevent cross-contamination. DNA was purified using a modified protocol based on the QIAamp DNA Mini Kit (Cat. No. 51306; QIAGEN, Hilden, Germany). Briefly, one disc was incubated in 180 µL of Buffer ATL at 85 °C for 10 min, followed by digestion with 20 µL Proteinase K (56 °C, 1 h) and treatment with 4 µL RNase A (37 °C, 5 min). After addition of Buffer AL and ethanol, the lysates were transferred to QIAamp spin columns. Column purification was carried out according to the manufacturer’s protocol with minor modifications designed to improve recovery from dried blood spots. At each wash step, buffers AW1 and AW2 were allowed to incubate on the column for 2 min prior to centrifugation at 15,000× *g*. The AW2 wash was performed twice, and centrifugation times were extended up to 5 min to ensure complete removal of residual wash buffer. DNA was eluted in two sequential steps of 25 µL Buffer AE, applied directly to the membrane without contact, incubated for 5 min at room temperature, and centrifuged to obtain a final eluate of 50 µL. Genomic DNA from cultured cell pellets was extracted in parallel using the NucleoSpin kit (Macherey-Nagel, Düren, Germany) following the manufacturer’s instructions. DNA concentration and purity were assessed using a NanoDrop 2000 spectrophotometer (Thermo Scientific, Waltham, MA, USA). Extraction blanks (filter paper discs without blood) were processed in parallel as negative controls. To verify successful extraction and overall integrity, 1 µL of each sample was first run on a 1% agarose gel with 3 µL loading buffer and visualized under UV illumination. 

DNA had been previously extracted from the paraffin-embedded tissues by the contributing pathology laboratories using standard protocols validated for formalin-fixed, paraffin-embedded (FFPE) material. Genomic DNA was isolated from 10-μm sections of paraffin-embedded tissues. Briefly, after overnight digestion with proteinase K, DNA was purified using an EZ1 BioRobot workstation and the EZ1 DNA Tissue Kit (QIAGEN, Hilden, Germany), according to the manufacturer’s instructions. In our laboratory, DNA concentration and purity were assessed using a NanoDrop 2000 spectrophotometer (Thermo Scientific, USA). To ensure consistency for downstream analyses, all samples were diluted to comparable concentrations (8–10 ng/µL). 

### 4.4. E1A Detection by PCR and Nested PCR

#### 4.4.1. DNA Quality Control

The integrity and quality of DNA extracted from Guthrie cards and paraffin-embedded tissues were first evaluated by PCR amplification of the human *β-actin* gene. Reactions were carried out in a final volume of 25 µL consisting of: 14.1 µL sterile water, 2.5 µL 10× buffer (Bioline, London, UK), 0.75 µL MgCl_2_ (Bioline), 2.5 µL dNTP mix (2 mM, Invitrogen/Ambion, Carlsbad, CA, USA), 0.5 µL forward primer (Sigma, St. Louis, MO, USA), 0.5 µL reverse primer (Sigma), 0.15 µL Taq polymerase (Bioline), and 4 µL DNA template. The primer sequences were: forward 5′-ACACTGTGCCCATCTACGAGG-3′, reverse 5′-AGGGGCCGGACTCGTCATACT-3′, designed using Oligo7 software (Molecular Biology Insights, Inc., Cascade, CO, USA). Amplification was performed in a Veriti thermal cycler (Applied Biosystems, Foster City, CA, USA) under the following conditions: initial denaturation at 94 °C for 2 min; 40 cycles of 94 °C for 20 s, 57 °C for 30 s, and 72 °C for 30 s; followed by a final extension at 72 °C for 4 min and hold at 4 °C. PCR products were analyzed by electrophoresis in 2% agarose gels and visualized under UV illumination. 

#### 4.4.2. Detection of Adenoviral *E1A*

For the detection of *E1A* sequences in Guthrie cards and paraffin samples, multiple PCR approaches were evaluated. Custom primers designed with Oligo7 software (Molecular Biology Insights, Inc., Cascade, CO, USA) were used to amplify different regions of the adenoviral *E1A* gene. All reactions were performed in a 25 µL volume containing 2.5 µL 10× SO_4_ buffer, 0.5 µL MgCl_2_ (100 mM), 0.5 µL dNTP mix (10 mM), 1 µL forward primer, 1 µL reverse primer, 0.1 µL SuperHot Taq polymerase (BIORON GmbH, Römerberg, Germany), 18.4 µL nuclease-free water, and 1 µL DNA template; however, when the DNA concentration was low (<30 ng/µL), the input volume was increased to 4 µL to enhance sensitivity. Positive (293 cells and template-spiked reaction for *E1A*) and negative controls (extraction blanks and no-template controls) were included in each experiment. Pre-PCR procedures (sample handling, DNA extraction, and reaction setup) and post-PCR procedures (amplification and product analysis) were performed in physically separated laboratory areas with dedicated equipment and consumables, using a unidirectional workflow, aerosol-resistant filter tips throughout, and routine decontamination with DNA-decontaminating agents (DNAZap) and UV irradiation. PCR products were analyzed by electrophoresis in 2% agarose gels and visualized under UV illumination. 

Standard PCR for Guthrie cards was initially performed with primers ADV989F (5′-TTAGATTATGTGGAGCACCCC-3′) and ADV1347R (5′-ACAGCTATCCGTACTACTATTGCAT-3′), targeting a 383 bp fragment of the *E1A* gene. Cycling conditions were: 94 °C for 2 min; 40 cycles of 94 °C for 20 s, annealing at 61 °C for 30 s, and 72 °C for 30 s; final extension at 72 °C for 4 min, hold at 4 °C. Standard PCR for paraffin samples used primers ADV888F (5′-TTTCTATGCCAAACCTTGTACCG-3′) and ADV1015R (5′-TAATGACAAGACCTGCAACCG-3′), yielding a 148 bp product designed to improve amplification from degraded DNA. Cycling conditions were identical, except for an annealing temperature of 60 °C. 

When single-round PCR was inconclusive, a nested PCR strategy was applied for both Guthrie cards and paraffin DNA. First-round amplification was performed with primers ADV829F (5′-GGAGCCGCCTCACCTTTC-3′) and ADV1131R (5′-CCCACCACTCTATCACCCACTG-3′), generating a 324 bp fragment. One microliter of this product was used as a template for the inner PCR with primers ADV888F and ADV1071R (5′-CAGGTCCTCATATAGCAAAGCGAAC-3′), producing a 208 bp fragment. Cycling conditions were: 94 °C for 2 min; 40 cycles of 95 °C for 12 s, annealing at 60–61 °C for 20–30 s depending on primer set, and 72 °C for 30 s; final extension at 72 °C for 4 min, hold at 4 °C. 

Extensive optimization was performed by adjusting MgCl_2_ and primer concentrations, annealing times and temperatures, and DNA input until reproducible amplification of the expected product was achieved with consistent controls. 

### 4.5. Sequencing of PCR Products

To confirm the presence of adenoviral *E1A* sequences, PCR-positive amplicons that yielded products of sufficient quality for sequencing were submitted for Sanger sequencing at the Sequencing Service of the University of Zaragoza. In total, 23 amplicons were sequenced, including 11 from Guthrie card samples and 12 from paraffin-derived samples. For each sample, 5 µL of PCR product along with the corresponding primers were provided. Cycle sequencing was performed using fluorescent dye terminators and resolved on an ALF-Express automated DNA sequencer (GE Healthcare, UK). Sequencing was performed bidirectionally whenever possible. Forward and reverse reads were inspected, trimmed, and assembled to generate consensus sequences. A representative non-redundant consensus sequence from the internal region of the *E1A* amplicon was used for final database comparison and GenBank submission, as the confirmed sequences were identical or nearly identical. Sequence similarity searches were performed using BLASTn and BLASTx through the NCBI BLAST web interface, corresponding to the BLAST+ suite version 2.17.0+. BLAST results were evaluated using accession numbers, percent identity, query coverage, and protein-level similarity to adenoviral E1A. 

### 4.6. Statistical Analysis

Comparisons of *E1A* detection frequencies between cases and controls in Guthrie cards and paraffin samples were performed using two-sided Fisher’s exact tests in GraphPad Prism 10.2 (GraphPad Software, Corp., San Diego, CA, USA). Associations between *E1A* detection and tumor type were evaluated using contingency table analysis (chi-square test) after grouping tumor categories to avoid sparse data in individual subgroups. Paired Guthrie card and paraffin tumor samples were compared using McNemar’s exact test. *p* value < 0.05 was considered statistically significant. 

## 5. Conclusions

This study shows that adenoviral *E1A* sequences can be recovered from long-stored neonatal Guthrie cards and from paraffin-embedded pediatric tumors using short-amplicon nested PCR despite variable DNA quality. To our knowledge, this work extends previous leukemia-focused neonatal studies to additional pediatric tumor types, broadening the scope of early-life adenoviral detection. *E1A* detection was observed in both neonatal and tumor-derived material, with a more pronounced difference between tumors and paraffin controls than between patient-derived and control Guthrie cards, and paired Guthrie–tumor analyses revealed only partial concordance. Together, these patterns indicate heterogeneous detection of adenoviral *E1A* across neonatal and tumor samples, without establishing a specific mechanistic model for viral persistence, reactivation, or oncogenic action. Because viral DNA was also found in control Guthrie cards and, less frequently, in paraffin controls, these data do not by themselves establish a causal role of adenovirus in childhood tumorigenesis. Accordingly, Guthrie cards emerge as a practical archival resource for reconstructing early-life viral contacts, aligning with Sustainable Development Goal 3 (Good Health and Well-Being) by supporting molecular strategies aimed at earlier identification of childhood cancers. 

Future work will require quantitative assays to distinguish low-level background detection from true enrichment, larger and more completely paired series to test temporal stability of viral DNA, and integrative genomic and epigenetic readouts to determine whether *E1A*-positive samples carry molecular signatures compatible with possible virus-associated chromatin perturbation. Such studies will be necessary to determine whether neonatal *E1A* detection represents incidental viral exposure, low-level or episodic persistence, or a biologically meaningful marker associated with childhood cancer risk. 

## Figures and Tables

**Table 1:** Summary of DNA yield and purity for Guthrie card and paraffin-embedded specimens used in this study. Values are expressed as mean and range for DNA concentration and A260/A280 ratio.

Sample	Total Samples	Mean DNA Concentration	Range	A260/A280 Mean	Range
Guthrie cards	86	15 ng/µL	9–21 ng/µL	1.75	1.68–1.92
Paraffin-embedded tissues	95	254 ng/µL	8–500 ng/µL	1.81	1.52–2.10

**Table 2:** PCR-based detection of *E1A* in Guthrie Cards, Paraffin Tumor Samples, and Controls.

Sample Type	Total Samples	*E1A*-Positive n (%)	*E1A*-Negative n, (%)
Guthrie card	54	23 (43%)	31 (57%)
Guthrie card (control)	32	11 (34%)	21 (66%)
Paraffin	75	31 (41%)	44 (59%)
Paraffin (control)	20	1 (5%)	19 (95%)

## Data Availability

The original contributions presented in this study are included in the article/Supplementary Materials. The representative adenoviral *E1A* consensus sequence has been deposited in GenBank under accession number PZ339933.

## Supplementary Materials

The following supporting information can be downloaded at: https://www.mdpi.com/article/10.3390/ijms27094047/s1.

## Abbreviations

The following abbreviations are used in this manuscript:

ALL	Acute Lymphoblastic Leukemia
ATRX	Alpha-thalassemia/mental retardation syndrome X-linked protein
ATL	Buffer ATL
AW1/AW2	Wash Buffers 1 and 2
BLAST	Basic Local Alignment Search Tool
BLASTn	Basic Local Alignment Search Tool nucleotide
BLASTx	Translated nucleotide–protein BLAST
bp	Base Pair
CBP	CREB-Binding Protein
CMV	Cytomegalovirus
CNS	Central Nervous System
C^+^/C^−^	Positive/Negative Control
Daxx	Death-domain associated protein
DBS	Dried Blood Spot
DNA	Deoxyribonucleic Acid
dNTP	Deoxynucleotide Triphosphate
*E1A*	Early region 1A
E2F	E2F transcription factor family
FFPE	Formalin-fixed paraffin-embedded
HAdV-C	Human mastadenovirus species C
HPV	Human papillomavirus
MDA	Multiple-displacement amplification National
NCBI	Center for Biotechnology Information
Rb	Retinoblastoma protein
SV40	Simian virus 40
