# Supplementary material for: Detection of Adenoviral E1A Gene in Guthrie Cards for Insights into Pediatric Cancer Origin
Source: Int J Mol Sci. 2026 Apr 30;27(9):4047. doi: 10.3390/ijms27094047 (PMC13163417; doi:10.3390/ijms27094047)
Supplement: Supplementary file 1 [file ijms-27-04047-s001.zip › ijms-4204416-supplementary.pdf]

Supplementary Materials

Detection of Adenoviral *E1A* Gene in Guthrie Cards for Insights into Pediatric Cancer Origin

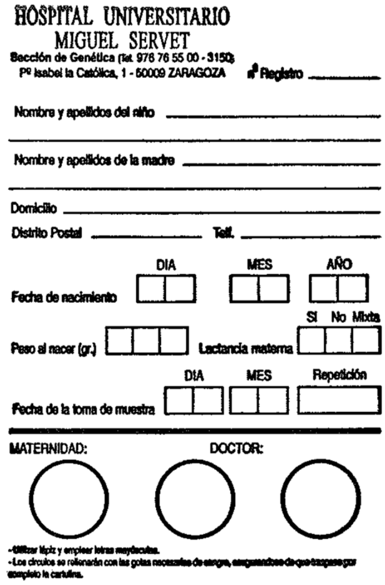

HOSPITAL UNIVERSITARIO
MIGUEL SERVET
Sección de Genética (tel. 976 76 55 00 - 3150)
Pº Isabel la Católica, 1 - 50009 ZARAGOZA

nº Registro ____________

Nombre y apellidos del niño ____________

Nombre y apellidos de la madre ____________

Domicilio ____________

Distrito Postal ____________ Telf. ____________

	DIA	MES	AÑO
Fecha de nacimiento			

Peso al nacer (gr.) | Lactancia materna: Si / No / Mixta

	DIA	MES	Repetición
Fecha de la toma de muestra			

MATERNIDAD: DOCTOR:

-Utilizar lápiz y emplear letras mayúsculas.
-Los círculos se rellenarán con las gotas [illegible] completo la cartulina.

Figure S1. Representative neonatal Guthrie card used for routine newborn screening.

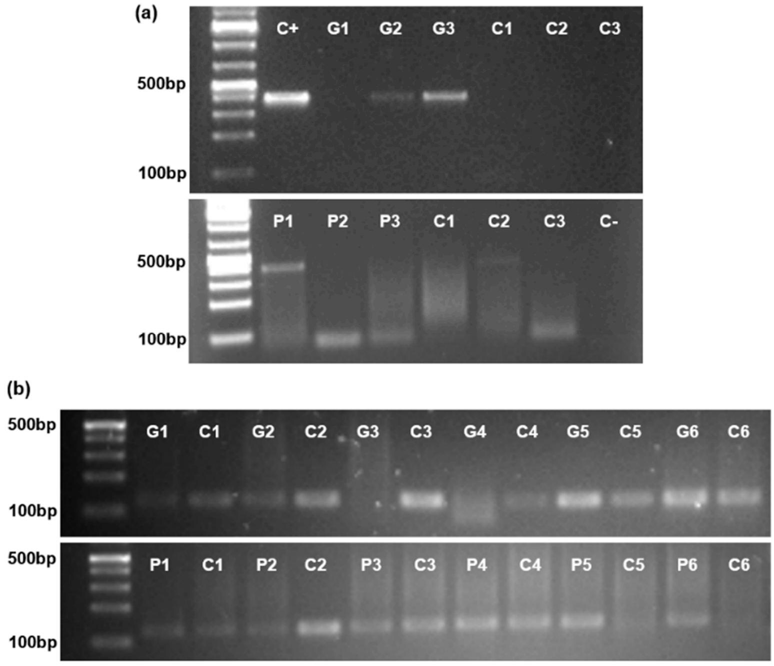

Figure S2. PCR amplification in Guthrie cards and paraffin-derived DNA. **(a)** Primers ADV989F and ADV1347R targeting a 383 bp fragment. **(b)** Primers ADV888F and ADV1015R yielding a 148 bp product. Representative samples are shown for both panels. Lanes labeled **G** correspond to Guthrie card samples (G1, G2, etc.), lanes labeled **P** to paraffin-derived samples (P1, P2, etc.) and lanes labeled **C** correspond to control samples for each sample type. C^+: positive control (293 cell DNA). C^-: water negative control.

Table S1. BLAST alignment results of consensus E1A sequences. Summary of top database hits obtained by BLAST analysis of the consensus E1A sequence derived from PCR-positive samples.

Accession	Description	Scientific Name	Per. ident	E value	Max Score	Query Cover
MK041230.1	Human mastadenovirus C isolate human/Shanxi-CHN/160/2001, complete genome	Human mastadenovirus C	100	5.00E-54	224	100%
OQ518339.1	Human adenovirus 108 isolate HAdV-C108/USA/2A4/2009, complete genome	Human adenovirus 108	100	5.00E-54	224	100%
PX146732.1	Human adenovirus 2 isolate hAdV-C2/Russia/KDA-RII-MH209691V/2024, complete genome	Human adenovirus 2	100	5.00E-54	224	100%
PP068613.1	Human adenovirus C108 isolate HAdV-C108/USA/10S6/2013, complete genome	Human adenovirus C108	100	5.00E-54	224	100%
MZ603080.1	MAG: Human mastadenovirus C isolate HAdV-5_RVAB genomic sequence	Human mastadenovirus C	100	5.00E-54	224	100%
PP386306.1	Human mastadenovirus C strain MBC001, complete genome	Human mastadenovirus C	100	5.00E-54	224	100%
PX146750.1	Human adenovirus 89 isolate hAdV-C89/Russia/SPE-RII-MH204046V/2024, complete genome	Human adenovirus 89	100	5.00E-54	224	100%
OQ518312.1	Human adenovirus 2 isolate HAdV-C2/USA/6H1/2010, complete genome	Human adenovirus 2	100	5.00E-54	224	100%
PP442595.1	Cloning vector pBWH-C2-RC, complete sequence	Cloning vector pBWH-C2-RC	100	5.00E-54	224	100%
PX146747.1	Human adenovirus 5 isolate hAdV-C5/Russia/KDA-RII-MH209690V/2024, complete genome	Human adenovirus 5	100	5.00E-54	224	100%
LC791175.1	Human mastadenovirus C Kobe190455 DNA, complete genome	Human mastadenovirus C	100	5.00E-54	224	100%
OQ518268.1	Human adenovirus 5 isolate HAdV-C5/USA/8Q8/2011, complete genome	Human adenovirus 5	100	5.00E-54	224	100%
OQ518342.1	Human adenovirus 108 isolate HAdV-C108/USA/6L10/2010, complete genome	Human adenovirus 108	100	5.00E-54	224	100%
OM112285.1	Human mastadenovirus C strain C2ONP01Cu1Nov2015, complete genome	Human mastadenovirus C	100	5.00E-54	224	100%
LC791119.1	Human mastadenovirus C Kobe180152 DNA, complete genome	Human mastadenovirus C	100	5.00E-54	224	100%
OQ518289.1	Human adenovirus 5 isolate HAdV-C5/USA/9T1/2012, complete genome	Human adenovirus 5	100	5.00E-54	224	100%
OM112284.1	Human mastadenovirus C strain C2ONP03Pr1Oct2016, complete genome	Human mastadenovirus C	100	5.00E-54	224	100%
MZ603084.1	MAG: Human mastadenovirus C isolate HAdV-1_RVAB genomic sequence	Human mastadenovirus C	100	5.00E-54	224	100%
OQ518341.1	Human adenovirus 89 isolate HAdV-C89/USA/7M3/2011, complete genome	Human adenovirus 89	100	5.00E-54	224	100%

PX146754.1	Human adenovirus 89 isolate hAdV-C89/Russia/KDA-RII-MH209689V/2024, complete genome	Human adenovirus 89	100	5.00E-54	224	100%
LC068717.1	Human adenovirus 6 DNA, nearly complete genome, strain: 1040264	Human adenovirus 6	100	5.00E-54	224	100%
LC068715.1	Human adenovirus 6 DNA, nearly complete genome, strain: 940162	Human adenovirus 6	100	5.00E-54	224	100%
OR777220.1	Human adenovirus 2 isolate HAdV-C2/USA/13R1/2017, complete genome	Human adenovirus 2	100	5.00E-54	224	100%
LC791155.1	Human mastadenovirus C Kobe190175 DNA, complete genome	Human mastadenovirus C	100	5.00E-54	224	100%
OR753135.1	Human adenovirus 89 isolate HAdV-C89/USA/8S6/2012, complete genome	Human adenovirus 89	100	5.00E-54	224	100%
PX146736.1	Human adenovirus 2 isolate hAdV-C2/Russia/KDA-RII-MH209688V/2024, complete genome	Human adenovirus 2	100	5.00E-54	224	100%
PV092666.1	Human adenovirus 108 isolate BJ-2024-1226/2021, complete genome	Human adenovirus 108	100	5.00E-54	224	100%
MH121080.1	Human mastadenovirus C strain 11C2, partial genome	Human mastadenovirus C	100	5.00E-54	224	100%
OR735212.1	Human adenovirus 89 isolate HAdV-C89/USA/6C1/2010, complete genome	Human adenovirus 89	100	5.00E-54	224	100%
OQ518350.1	Human adenovirus 2 isolate HAdV-C2/USA/5R10/2010, complete genome	Human adenovirus 2	100	5.00E-54	224	100%
JX173079.1	Human adenovirus C strain human/ARG/A15932/2002/2[P2H2F2], complete genome	Human mastadenovirus C	100	5.00E-54	224	100%
MH121113.1	Human mastadenovirus C strain 46C6, partial genome	Human mastadenovirus C	100	5.00E-54	224	100%
PX146755.1	Human adenovirus 89 isolate hAdV-C89/Russia/KDA-RII-MH209693V/2024, complete genome	Human adenovirus 89	100	5.00E-54	224	100%
OR777154.1	Human adenovirus 5 isolate HAdV-C5/USA/1B6/2020, complete genome	Human adenovirus 5	100	5.00E-54	224	100%
MH121079.1	Human mastadenovirus C strain 10C2, partial genome	Human mastadenovirus C	100	5.00E-54	224	100%
OR876398.1	Human adenovirus 5 isolate HAdV-C5/USA/7D10/2010, complete genome	Human adenovirus 5	100	5.00E-54	224	100%
PP068615.1	Human adenovirus 2 isolate HAdV-C2/USA/5R6/2010, complete genome	Human adenovirus 2	100	5.00E-54	224	100%
OR735211.1	Human adenovirus 1 isolate HAdV-C1/USA/6B1/2010, complete genome	Human adenovirus 1	100	5.00E-54	224	100%
OR753117.1	Human adenovirus 2 isolate HAdV-C2/USA/6C6/2010, complete genome	Human adenovirus 2	100	5.00E-54	224	100%
OR777218.1	Human adenovirus 5 isolate HAdV-C5/USA/13O1/2017, complete genome	Human adenovirus 5	100	5.00E-54	224	100%
OR735190.1	Human adenovirus 1 isolate HAdV-C1/USA/10B7/2012, complete genome	Human adenovirus 1	100	5.00E-54	224	100%
LC791182.1	Human mastadenovirus C Kobe190517 DNA, complete genome	Human mastadenovirus C	100	5.00E-54	224	100%

PP786308.1	Human mastadenovirus C isolate CHN/BJ/86413/2017, complete genome	Human mastadenovirus C	100	5.00E-54	224	100%
AY339865.1	Human adenovirus C serotype 5, complete genome	Human mastadenovirus C	100	5.00E-54	224	100%
MH121082.1	Human mastadenovirus C strain 13C1, partial genome	Human mastadenovirus C	100	5.00E-54	224	100%
OQ518335.1	Human adenovirus 2 isolate HAdV-C2/USA/6A4/2010, complete genome	Human adenovirus 2	100	5.00E-54	224	100%
PX146751.1	Human adenovirus 89 isolate hAdV-C89/Russia/PSK-RII-MH204073V/2023, complete genome	Human adenovirus 89	100	5.00E-54	224	100%
OQ834914.1	Human mastadenovirus C isolate sewage/Tianjin-CHN/120204/2021, complete genome	Human mastadenovirus C	100	5.00E-54	224	100%
PQ336898.1	Human adenovirus 89 isolate HAdV_WGS-C/KENKLF_P_HFK1037/2023, partial genome	Human adenovirus 89	100	5.00E-54	224	100%
PX146731.1	Human adenovirus 2 isolate hAdV-C2/Russia/SPE-RII-MH209709V/2024, complete genome	Human adenovirus 2	100	5.00E-54	224	100%
OR735195.1	Human adenovirus 1 isolate HAdV-C1/USA/9P3/2012, complete genome	Human adenovirus 1	100	5.00E-54	224	100%
OR735197.1	Human adenovirus 89 isolate HAdV-C89/USA/7I7/2011, complete genome	Human adenovirus 89	100	5.00E-54	224	100%
OP555463.1	Human mastadenovirus C isolate P-10, complete genome	Human mastadenovirus C	100	5.00E-54	224	100%
PP079214.1	Human adenovirus 2 isolate HAdV-C2/USA/9C10/2012, complete genome	Human adenovirus 2	100	5.00E-54	224	100%
PV092655.1	Human adenovirus 108 isolate WZ-2024-1221/2019, complete genome	Human adenovirus 108	100	5.00E-54	224	100%
HQ003817.1	Human adenovirus C strain human/RUS/16700/2001/57[P1H57F6], complete genome	Human mastadenovirus C	100	5.00E-54	224	100%
BK066413.1	MAG TPA_asm: Adenovirus homo5 isolate Adeno5 genomic sequence	Adenovirus homo5	100	5.00E-54	224	100%
MH121111.1	Human mastadenovirus C strain 44C2, partial genome	Human mastadenovirus C	100	5.00E-54	224	100%
OR735189.1	Human adenovirus 1 isolate HAdV-C1/USA/8B5/2011, complete genome	Human adenovirus 1	100	5.00E-54	224	100%
OQ518291.1	Human adenovirus 89 isolate HAdV-C89/USA/5L6/2009, complete genome	Human adenovirus 89	100	5.00E-54	224	100%
OR735191.1	Human adenovirus 89 isolate HAdV-C89/USA/8C3/2011, complete genome	Human adenovirus 89	100	5.00E-54	224	100%
MZ151861.1	Human mastadenovirus C strain HAdVC/Novosibirsk/7.134V/2019, complete genome	Human mastadenovirus C	100	5.00E-54	224	100%
OU501480.1	Human adenovirus 2 isolate ADVJA-23-BE genome assembly, chromosome: ADVJA-23-BE	Human adenovirus 2	100	5.00E-54	224	100%
OR777173.1	Human adenovirus 5 isolate HAdV-C5/USA/7D6/2018, complete genome	Human adenovirus 5	100	5.00E-54	224	100%
MH121093.1	Human mastadenovirus C strain 24C2, partial genome	Human mastadenovirus C	100	5.00E-54	224	100%

OQ518319.1	Human adenovirus 5 isolate HAdV-C5/USA/5N1/2009, complete genome	Human adenovirus 5	100	5.00E-54	224	100%
LC791147.1	Human mastadenovirus C Kobe190036 DNA, complete genome	Human mastadenovirus C	100	5.00E-54	224	100%
OQ518263.1	Human adenovirus 5 isolate HAdV-C5/USA/10B3/2012, complete genome	Human adenovirus 5	100	5.00E-54	224	100%
OM112289.1	Human mastadenovirus C strain C2ONP02Cu1Jan2016, complete genome	Human mastadenovirus C	100	5.00E-54	224	100%
OQ834917.1	Human mastadenovirus C isolate sewage/Tianjin-CHN/110103/2021, complete genome	Human mastadenovirus C	100	5.00E-54	224	100%
OQ518285.1	Human adenovirus 2 isolate HAdV-C2/USA/5S1/2010, complete genome	Human adenovirus 2	100	5.00E-54	224	100%
OR777203.1	Human adenovirus 89 isolate HAdV-C89/USA/12S1/2016, complete genome	Human adenovirus 89	100	5.00E-54	224	100%
MK041226.1	Human mastadenovirus C isolate human/Shanxi-CHN/122/2012, complete genome	Human mastadenovirus C	100	5.00E-54	224	100%
PX146720.1	Human adenovirus 1 isolate hAdV-C1/Russia/LIP-RII-MH149917V/2023, complete genome	Human adenovirus 1	100	5.00E-54	224	100%
OM112286.1	Human mastadenovirus C strain C2ONP07Pr1May2018, complete genome	Human mastadenovirus C	100	5.00E-54	224	100%
OR753138.1	Human adenovirus 1 isolate HAdV-C1/USA/8I7/2011, complete genome	Human adenovirus 1	100	5.00E-54	224	100%
PX146729.1	Human adenovirus 2 isolate hAdV-C2/Russia/PER-RII-MH148396V/2023, complete genome	Human adenovirus 2	100	5.00E-54	224	100%
OR735180.1	Human adenovirus 89 isolate HAdV-C89/USA/6J6/2010, complete genome	Human adenovirus 89	100	5.00E-54	224	100%
MH121086.1	Human mastadenovirus C strain 17C2, partial genome	Human mastadenovirus C	100	5.00E-54	224	100%
OR777157.1	Human adenovirus 5 isolate HAdV-C5/USA/1G2/2019, complete genome	Human adenovirus 5	100	5.00E-54	224	100%
OQ834910.1	Human mastadenovirus C isolate sewage/Tianjin-CHN/030101/2022, complete genome	Human mastadenovirus C	100	5.00E-54	224	100%
OQ518305.1	Human adenovirus 5 isolate HAdV-C5/USA/5P3/2010, complete genome	Human adenovirus 5	100	5.00E-54	224	100%
MH121094.1	Human mastadenovirus C strain 25C5, partial genome	Human mastadenovirus C	100	5.00E-54	224	100%
AY147066.1	Human adenovirus type 5 E1A protein gene, complete cds	Human adenovirus 5	100	5.00E-54	224	100%
MH629744.1	Human adenovirus 5 isolate ATCC-VR-5 289R (E1A), 243R (E1A), 19K (E1B), and 55K (E1B) genes, complete cds	Human adenovirus 5	100	5.00E-54	224	100%
MT277585.1	Mutant Human adenovirus 2 isolate HAdV-C2-dE3B-CMV-GFP, complete sequence	Human adenovirus 2	100	5.00E-54	224	100%
AY601635.1	Human adenovirus type 5 strain NHRC Ad5FS 7151, complete genome	Human adenovirus 5	100	5.00E-54	224	100%

OQ518337.1	Human adenovirus 108 isolate HAdV-C108/USA/8J10/2011, complete genome	Human adenovirus 108	100	5.00E-54	224	100%
OQ518302.1	Human adenovirus 89 isolate HAdV-C89/USA/7F2/2010, complete genome	Human adenovirus 89	100	5.00E-54	224	100%
OR735183.1	Human adenovirus 5 isolate HAdV-C5/USA/3M4/2009, complete genome	Human adenovirus 5	100	5.00E-54	224	100%
PX146735.1	Human adenovirus 2 isolate hAdV-C2/Russia/KDA-RII-MH209686V/2024, complete genome	Human adenovirus 2	100	5.00E-54	224	100%
OR876399.1	Human adenovirus 89 isolate HAdV-C89/USA/7F1/2010, complete genome	Human adenovirus 89	100	5.00E-54	224	100%
OQ518318.1	Human adenovirus 108 isolate HAdV-C108/USA/8K7/2011, complete genome	Human adenovirus 108	100	5.00E-54	224	100%
PX146749.1	Human adenovirus 57 isolate hAdV-C57/Russia/SPE-RII-MH204036V/2024, complete genome	Human adenovirus 57	100	5.00E-54	224	100%
OR777171.1	Human adenovirus 89 isolate HAdV-C89/USA/6R10/2010, complete genome	Human adenovirus 89	100	5.00E-54	224	100%
OR753106.1	Human adenovirus 89 isolate HAdV-C89/USA/5S2/2010, complete genome	Human adenovirus 89	100	5.00E-54	224	100%
LC791148.1	Human mastadenovirus C Kobe190044 DNA, complete genome	Human mastadenovirus C	100	5.00E-54	224	100%
MH121101.1	Human mastadenovirus C strain 33C2, partial genome	Human mastadenovirus C	100	5.00E-54	224	100%
OR735181.1	Human adenovirus 1 isolate HAdV-C1/USA/7L7/2011, complete genome	Human adenovirus 1	100	5.00E-54	224	100%
KF268127.1	Human adenovirus C strain human/USA/CL_42/1988/5[P5H5F5], complete genome	Human mastadenovirus C	100	5.00E-54	224	100%

Original assembled Consensus Sequence

GAAGAGGGTGAGGAGTTTGTGTTAGATTATGTGGAGCACCCCGGGCACGGTTGCAGGTCTGAAGAGGGTGAGGAGTTTGTGTTAGATTATGT
GGAGCACCCCGGGCACGGTTGCAGGTCTTGTCATTATCACCGGAGGAATACGGGGGACCCAGATATTATGTGTTCGCTTTGCTATATGTGTC
ATTATCACCGGAGGAATACGGGGGACCCAGATATTATGTGTTCGCTTTGCTATATG

Trimmed Consensus Sequence

TGAAGAGGGTGAGGAGTTTGTGTTAGATTATGTGGAGCACCCCGGGCACGGTTGCAGGTCTTGTCATTATCACCGGAGGAATACGGGGGACC
CAGATATTATGTGTTCGCTTTGCTATATG

Table S2. Distribution of pediatric tumor types included in the study.

Central Nervous System (CNS) Tumors	Medulloblastoma, astrocytoma, retinoblastoma, low-grade astrocytoma, neurofibromatosis, pineoblastoma, craniopharyngioma, desmoplastic medulloblastoma, pilocytic astrocytoma, ganglioma, pilomyxoid astrocytoma, classical ependymoma, nodular ganglioneuroblastoma, infantile desmoplastic ganglioglioma, anaplastic medulloblastoma
Lymphomas	High-grade B-cell lymphoma (Burkitt type), lymphoblastic lymphoma, anaplastic large cell lymphoma, cutaneous B-cell lymphoma, classical Hodgkin lymphoma, pheochromocytoma, metanephric adenoma, metastasis of neuroblastoma / ganglioneuroblastoma, congenital mesoblastic nephroma
Soft Tissue Tumors	Spindle cell rhabdomyosarcoma, osteoblastic osteosarcoma, desmoplastic tumor, atypical Ewing sarcoma, rhabdoid tumor, neurilemmoma, renal sarcoma
Renal Tumors	Wilms tumor, cystic Wilms tumor
Hepatic Tumors	Hepatoblastoma, hepatocarcinoma, well-differentiated hepatocarcinoma
Germ Cell Tumors	Yolk sac (endodermal sinus tumor), juvenile granulosa cell tumor
Other Tumors	Inflammatory myofibroblastic tumor (lung), pilomatrixoma (cutaneous), ovary, testis, mediastinum, skin
